# Comprehensive Utilization of Marine Microalgae for Enhanced Co-Production of Multiple Compounds

**DOI:** 10.3390/md18090467

**Published:** 2020-09-16

**Authors:** Ruijuan Ma, Baobei Wang, Elvis T. Chua, Xurui Zhao, Kongyong Lu, Shih-Hsin Ho, Xinguo Shi, Lemian Liu, Youping Xie, Yinghua Lu, Jianfeng Chen

**Affiliations:** 1 Technical Innovation Service Platform for High Value and High Quality Utilization of Marine Organism, Fuzhou University, Fuzhou 350108, China; rjma@fzu.edu.cn (R.M.); YP1905@fzu.edu.cn (K.L.); stephen6949@hit.edu.cn (S.-H.H.); xshi@fzu.edu.cn (X.S.); lmliu@fzu.edu.cn (L.L.); 2 Fujian Engineering and Technology Research Center for Comprehensive Utilization of Marine Products Waste, Fuzhou University, Fuzhou 350108, China; 3 Fuzhou Industrial Technology Innovation Center for High Value Utilization of Marine Products, Fuzhou University, Fuzhou 350108, China; 4 College of Oceanology and Food Science, Quanzhou Normal University, Quanzhou 362000, China; baobeiw@qztc.edu.cn; 5 Algae Biotechnology Laboratory, School of Agriculture and Food Sciences, The University of Queensland, Brisbane, QLD 4072, Australia; e.chua@uq.net.au; 6 Department of Chemical and Biochemical Engineering, College of Chemistry and Chemical Engineering, Xiamen University, Xiamen 361005, China; 20620190154099@stu.xmu.edu.cn (X.Z.); ylu@xmu.edu.cn (Y.L.); 7 State Key Laboratory of Urban Water Resource and Environment, School of Environment, Harbin Institute of Technology, Harbin 150090, China

**Keywords:** marine microalgae, comprehensive utilization, multiple compounds, co-production, economic feasibility

## Abstract

Marine microalgae are regarded as potential feedstock because of their multiple valuable compounds, including lipids, pigments, carbohydrates, and proteins. Some of these compounds exhibit attractive bioactivities, such as carotenoids, ω-3 polyunsaturated fatty acids, polysaccharides, and peptides. However, the production cost of bioactive compounds is quite high, due to the low contents in marine microalgae. Comprehensive utilization of marine microalgae for multiple compounds production instead of the sole product can be an efficient way to increase the economic feasibility of bioactive compounds production and improve the production efficiency. This paper discusses the metabolic network of marine microalgal compounds, and indicates their interaction in biosynthesis pathways. Furthermore, potential applications of co-production of multiple compounds under various cultivation conditions by shifting metabolic flux are discussed, and cultivation strategies based on environmental and/or nutrient conditions are proposed to improve the co-production. Moreover, biorefinery techniques for the integral use of microalgal biomass are summarized. These techniques include the co-extraction of multiple bioactive compounds from marine microalgae by conventional methods, super/subcritical fluids, and ionic liquids, as well as direct utilization and biochemical or thermochemical conversion of microalgal residues. Overall, this review sheds light on the potential of the comprehensive utilization of marine microalgae for improving bioeconomy in practical industrial application.

## 1. Introduction

Marine microalgae are photosynthetic microorganisms that use light and inorganic nutrients to produce various compounds, including lipids, pigments, carbohydrates, and proteins [1]. Some of these compounds exhibit attractive bioactivities [2]. For example, the carotenoid lutein has the human health benefits of ameliorating cardiovascular diseases, cancers, and age-related macular degeneration (AMD) [3]. Eicosapentaenoic acid (EPA) and docosahexaenoic acid (DHA), two ω-3 polyunsaturated fatty acids, can support the cardiovascular, brain, and eye systems, and have anti-inflammatory, antibiotic, and anticancer activities in humans [4,5]. Calcium spirulan, a polysaccharide, has antiviral activities [6], as well as the functions in inhibition of tumor invasion and metastasis [7]. In addition, some protein-derived peptides from marine microalgae were found to have bioactivity, such as antioxidative and antihypertensive properties [8]. Thus, these bioactive compounds can be used in pharmaceutical, nutraceutical, and cosmeceutical. However, the contents of these bioactive compounds are normally at low levels, and thus, the production costs are quite high. Aside from the bioactive compounds, the components of marine microalgae contain a large amount of high-nutrition and high-energy compounds, which can be used in food/feed and biofuel industries [9]. Hence, the comprehensive utilization of marine microalgae by using most components of biomass for the co-production of multiple compounds instead of sole products can be an efficient way to reduce the production cost of bioactive compounds and improve production efficiency. This includes enhancing the production and biorefinery abilities to obtain multiple compounds from marine microalgae [10,11].

Marine microalgae are considered as versatile cellular factories for valued products [4]. A specific marine microalgae species can be used as a feedstock for many different bio-compounds. For instance, *Phaeodactylum tricornutum* is a good candidate for fucoxanthin and EPA [12], *Dunaliella salina* can be used to produce proteins and carotenoids [13], and *Spirulina* sp. Can accumulate a large amount of carbonic anhydrase, C-phycocyanin, and allophycocyanin [14]. However, there are only a few studies on the co-production of multiple compounds by marine microalgae. The potential of using specific microalgae for multiple compounds production still needs to be explored. Metabolisms of lipids, pigments, carbohydrates, and proteins, are in an interactive metabolic network. Hence, it is possible to tune the marine microalgae to accumulate two or more target metabolites by modulating the metabolic network under different cultivation conditions, adding value to marine microalgae production.

Moreover, complete utilization of all microalgal components rather than extracting only one product is another effective way to improve the economics of the production. Some compounds (such as fatty acid and lutein) can be simultaneously extracted from microalgal biomass, due to the similar physicochemical properties, such as polarity, hydrophobicity, solubility, and molecular weight [15]. Additionally, stepwise extraction of lipids, pigments, carbohydrates, and proteins from biomass has been performed in marine microalgae [11,16]. Previous studies mainly targeted single product, and microalgal residues were not completely used; but rather were discarded as wastes, which were still rich in valuable compounds [17,18]. Currently, the utilization of microalgal residues to increase the economic feasibility of marine microalgae production has been gaining attention worldwide.

This paper reviews the main valuable compounds in marine microalgae and their interaction in the metabolic network, summarizes current studies on the potential application of co-production of multiple compounds under various cultivation conditions and strategies, and revisits the biorefinery processes for integral use of microalgal biomass. The aim is to shed light on efficient ways to comprehensively utilize marine microalgae for improving bio-compound production.

## 2. Metabolic Network of Marine Microalgal Metabolites

The main components in marine microalgae are carbohydrates, proteins, lipids, and pigments. However, the contents of each compound in microalgal species are different. In addition, some marine microalgae can produce unique metabolites that are not found in other species. Thus, the potential of multi-compound co-production is species-specific. Table 1 shows different marine microalgae species that have the potential to produce multiple compounds.

Marine microalgal carbohydrates are mainly present as cellulose in the cell wall and starch in the plastid [32]. Monosaccharides are the basic components of carbohydrates in microalgae, which consist of glucose, galactose, mannose, rhamnose, arabinose, xylose, ribose, and fucose [33]. Among them, glucose is the major monosaccharide in microalgae. The biosynthesis of carbohydrate initiates from carbon dioxide (CO_2_) fixation through the Calvin cycle by ribulose-1, 5-bisphosphate carboxylase oxygenase (Rubisco) [34]. The product glyceraldehyde-3-phosphate (G3P), derived from 3-phosphoglyceraldehyde (3PGA), is subsequently catalyzed into monosaccharides, such as glucose, which are then converted into polysaccharides, such as cellulose and starch [32,35]. It is worth noting that the biosynthesis of carbohydrates and lipids competes for the same bulk of CO_2_ fixation, and the accumulation of storage carbohydrates consumes less energy than lipids per carbon basis [35]. Hence, storage carbohydrates are deemed to be short-term energy reserve, while lipids can store more available energy, thus are used for long-term energy storage [36,37,38,39].

The protein turnover rates in marine microalgae are comparable to that of higher plants [40], indicating the potential application of marine microalgae as protein feedstock. Amino acids, the main component of proteins, are derived from five intermediate products of glycolysis and citric acid cycle. G3P acts as an initial substrate for 20 amino acids [41]. Subsequently, 3-phosphoglyceric acid is catalyzed into serine, followed by cysteine and glycine; phosphoenolpyruvate is transferred into phenylalanine, tryptophan, and tyrosine; pyruvate is converted into alanine, valine, and leucine; oxaloacetate is catalyzed into aspartate, asparagine, lysine, methionine, threonine, and isoleucine; and α-ketoglutarate is catalyzed into glutamate, glutamine, histidine, proline, and arginine [41].

Microalgal lipids are considered as potential feedstock for lipid-based biodiesel [42]. In addition, some marine microalgae can synthesize omega-3 polyunsaturated fatty acids (ω-3PUFAs), such as EPA and DHA, which can be used in the pharmaceutical industry [43]. For lipid biosynthesis, acetyl-CoA, derived from pyruvate (a product of G3P), is catalyzed into malonyl-CoA by acetyl-CoA carboxylase (ACC), which is the first committed step of fatty acid synthesis [44,45]. Fatty acids are derived from the fatty acid synthesis (FAS) pathway, followed by the desaturase/elongase pathway or polyketide synthase (PKS) pathway to produce polyunsaturated fatty acids (PUFAs) [42]. Later, free fatty acids are catalyzed into triacylglycerol (TAG, main storage lipid in microalgae), by the glycerolipid pathway in both endoplasmic reticulum and chloroplasts [42]. To be noted, except for interaction with carbohydrate metabolism [46], lipid metabolism is closely connected to protein metabolism via the nitrogen metabolic pathway [47].

Chlorophylls, carotenoids, and phycobilins are three major types of pigments found in marine microalgae [48]. Among them, phycobilins are normally bound to protein, and then form phycobiliproteins (including blue phycocyanin, light blue allophycocyanin, purple phycoerythrin, and orange phycoerythrocyanin) [49]. Chlorophylls and phycobilins are biosynthesized from δ-aminolevulinic acid, which is derived from glutamate and is subsequently catalyzed into protoporphyrin IX [50,51]. On the other hand, the biosynthesis of carotenoids initiates from isopentenyl pyrophosphate (IPP) and its isomer dimethylallyl diphosphate (DMAPP) [52]. IPP is derived from acetyl-CoA by cytosolic mevalonic acid (MVA) pathway, or pyruvate and G3P by plastidic 2-C-methyl-D-erythritol-4-phosphate (MEP) pathway [53,54]. The intermediate of carotenoids biosynthesis, geranylgeranyl pyrophosphate, is another precursor of chlorophylls [51].

**Figure 1:**
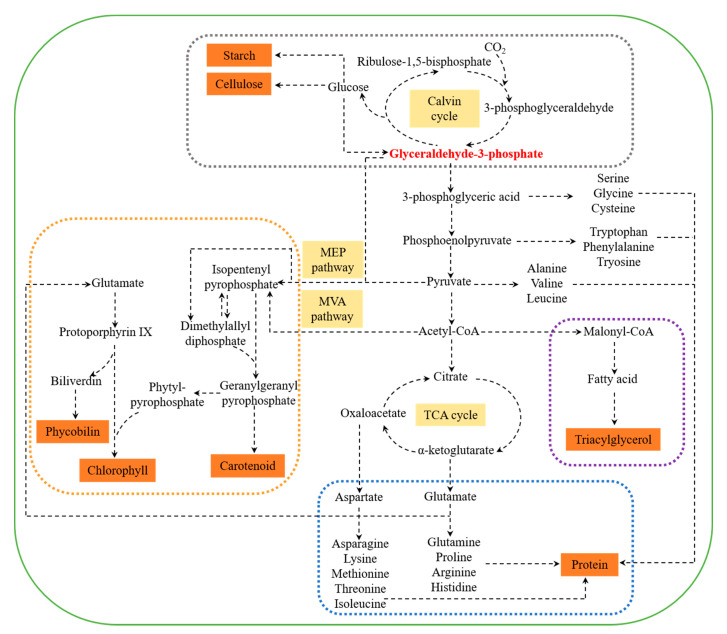
Metabolic network of carbohydrates, proteins, lipids, and pigments in marine microalgae. The grey box denotes the carbohydrate biosynthesis pathway; the blue box denotes the protein biosynthesis pathway; the purple box denotes the lipid biosynthesis pathway; and the orange box denotes the pigment biosynthesis pathway. MEP, 2-C-methyl-D-erythritol-4-phosphate; MVA, mevalonic acid; CoA, coenzyme A; TCA: tricarboxylic acid.

Thus, it can be confirmed that the biosynthesis of carbohydrates, proteins, lipids, and pigments are highly interconnected in the metabolic network by G3P (Figure 1). In current years, several novel techniques have been applied in microalgae to shift metabolic networks for enhanced target metabolite production. The rapidly developed omics technologies are helping to unlock transcription factors, cell cycle regulators, cell signaling, and metabolite biosynthesis in microalgal cells [55]. Concomitantly, genetic engineering technologies, such as RNA interference, gene knockout, and gene overexpression, can be used to modulate metabolic pathways and improve the biosynthesis of target metabolites. For example, a transcription factor NobZIP1 was identified from *Nannochloropsis oceanica* based on transcriptomic analysis, and then overexpression was performed for concurrent lipid overproduction and secretion [56]. RNAi-mediated silencing of a pyruvate dehydrogenase kinase (PDK) led to enhanced TAG biosynthesis in *N. salina*, and transcriptional analysis revealed that carbon metabolic flux diverted towards TAG synthesis by *PDK* gene knockdown [57]. On the other hand, the redirection of metabolism can be achieved by shifting trophic modes. It was shown that metabolic flux switched from the biosynthesis of starch to lipid when the cultivation condition shifted from heterotrophy into photoautotrophy in *Chlorella* spp. [58]. Moreover, modification of cultivation conditions, including environmental and nutrient conditions, is a simple way to change the metabolism direction for enhanced production of target metabolites. For example, metabolic flux shifted from lipid degradation into lipid biosynthesis under nitrogen starvation condition, leading to enhanced lipid accumulation in *Tetraselmis* sp. M8 [59]. Likewise, metabolic flux was channeled into carotenoid and TAG biosynthesis under high light conditions, resulting in an increase in carotenoid and TAG accumulation in *D. salina* [60]. Hence, modulation of cultivation conditions can be an easy and efficient approach to achieve the co-production of multiple target compounds.

## 3. Co-Production of Multiple Compounds in Marine Microalgae under Various Cultivation Conditions

Marine microalgal metabolites can be basically categorized into primary and secondary metabolites. Primary metabolites mainly include carbohydrates, lipids, proteins, and photosynthetic pigments, while secondary metabolites consist mainly of carotenoids, phytosterols, and phenolic compounds [61]. In general, primary metabolites are produced as a result of cell growth, development, and reproduction; however, secondary metabolites are uniquely accumulated to relieve cellular injuries under stress conditions. To be noted, some carotenoids, such as lutein [62] and fucoxanthin [26], are the components of the light-harvesting complex for photosynthesis and photo-protection, and thus, can be considered primary metabolites. A special example is β-carotene, which can be primary or secondary carotenoids depending on species [63]. On the other hand, microalgal lipids can be mainly divided into membrane lipids (consist of polar lipids) and storage lipids (consist of neutral lipids mainly in the form of TAG) [64]. Similarly, carbohydrates can be divided into structural carbohydrates and storage carbohydrates (such as starch, glycogen, and glucan) [34]. Generally, the membrane lipids and structural carbohydrates are related to cell growth, while storage lipids and carbohydrates accumulation are enhanced under stress conditions.

### 3.1. Co-Production of Multiple Compounds under Different Environmental Conditions

#### 3.1.1. Light Intensity

Light is a crucial factor for cell growth and metabolites accumulation in phototrophic marine microalgae. Generally, the accumulation of primary metabolites, such as membrane lipids and structural carbohydrates, are consistent with cell growth. Under high light conditions, storage lipids and carbohydrates are accumulated and become the bulk of lipids and carbohydrates. It was found that both lipids and carbohydrates contents increased with the increase in light intensity from 50 to 100 μmol/m^2^/s in *Neochloris oleoabundans* [38]. However, in some microalgae, available carbon is utilized to form lipids, but not starch under higher light intensity or longer time treatment [38,39]. This shift may be because the biosynthetic pathways of carbohydrates and lipids compete for the same precursor, and lipids can store more available energy than carbohydrates. Thus, microalgal cells prefer to accumulate lipids instead of carbohydrates for long-term storage under stressed conditions [65]. In addition, the low light intensity is optimal for primary carotenoids accumulation, such as lutein [62] and fucoxanthin [26]. However, the content of secondary carotenoids like β-carotene and zeaxanthin were enhanced significantly under high light conditions [66]. Similar to primary carotenoids, phycobiliproteins accumulation increased under low light intensity, while chlorophyll accumulation was consistent with cell growth [67].

Thus, it has been established that primary carotenoids and phycobiliproteins increase under low light intensity; structural carbohydrates, proteins, membrane lipids, and chlorophylls enhance under optimal light intensity for cell growth; while storage lipids and secondary carotenoids increase under high light conditions. This phenomenon can be part of the strategy in the co-production of multiple compounds. For example, in *N. oceanica*, the level of total lipids and secondary carotenoids (e.g., β-carotene, zeaxanthin, astaxanthin, and canthaxanthin) significantly increased under high light condition, when compared to that of the low light condition [68]. Additionally, the highest content of DHA and fucoxanthin were obtained at a low light intensity of 30 μmol/m^2^/s, and declined with the increase in light intensity to 120 μmol/m^2^/s in *Isochrysis* [27].

#### 3.1.2. Temperature

Temperature is regarded as one of the critical factors for marine microalgal growth and biochemical composition due to its role in cytoplasmic viscosity, photo-inhibition, and enzymatic activity [69]. In general, protein is accumulated as the cell growth progresses with respect to the variation in temperature, due to the structural and catalytic functions of protein [70]. However, the effect of temperature on carbohydrate accumulation is species-specific [34]. For example, in *Spirulina* sp., carbohydrate content increased with the rise in temperature from 25 to 40 °C, even though the optimal temperature for cell growth was 30 °C [70]. Nevertheless, in the diatom *Chaetoceros* cf. *wighamii*, biomass and carbohydrate contents decreased with the increase in temperature from 25 to 30 °C [71]. Lipid accumulation showed an opposite trend with cell growth, which was higher under extremely low or high temperatures [70]. This trend may be attributed to the increase in the accumulation of storage lipids at adverse temperatures. Noticeably, due to the changes in the cell membrane, the ratio of saturated and unsaturated fatty acids alters under different temperatures, as was observed in *N. oceanica* BR2 [72]. Unsaturated fatty acid content increases to maintain membrane fluidity, while saturated fatty acid content decreases under low temperatures [73]. In addition, the accumulation of secondary carotenoids such as zeaxanthin increases under relatively high temperatures [74], while primary carotenoids, like lutein, are enhanced under low temperatures [75]. In addition, phycobiliprotein accumulation is improved under relatively low temperatures [76].

The abovementioned studies show that temperature can be used as a parameter for the co-production of various metabolites. It can be concluded that relatively low temperatures is desirable for the biosynthesis of unsaturated fatty acids, primary carotenoids, and phycobiliproteins; optimal temperature for cell growth is desirable for the accumulation of proteins; relatively high temperatures is desirable for secondary carotenoids; and excessive low or high temperatures is desirable for storage lipids accumulation.

#### 3.1.3. pH

The pH is also considered to be a critical parameter in marine microalgal growth and metabolism. Most microalgae prefer to grow at neutral pH conditions, while some microalgae, such as *Spirulina*, are alkali-tolerant [70]. In general, chlorophylls and proteins contents are highest at optimal pH for cell growth, while the accumulation of storage carbohydrates and lipids showed the opposite trend with biomass [70,77]. This may be due to the enhanced storage carbohydrates and lipids accumulation under adverse pH conditions. On the other hand, high pH changes the NH_4_^+^/NH_3_ buffer system and induces free ammonia content in the culture, which is particularly toxic for the photosynthetic apparatus, affecting the accumulation of primary metabolites, such as chlorophylls and proteins [77,78]. In addition, the optimal pH for the accumulation of carotenoids [79] and phycobiliproteins [80] are consistent with cell growth, indicating that the optimal temperature is crucial for the biosynthesis of pigments. Hence, optimal pH for cell growth is suitable for chlorophylls, proteins, and carotenoids accumulation, while slightly low or high pH can enhance the accumulation of storage carbohydrates and lipids.

#### 3.1.4. Salinity

Marine microalgae are normally isolated from saline or brackish environments, and some of them can grow in a wide range of salinity. Optimal salinity condition is essential for microalgal growth and accumulation of primary metabolites, whereas extreme salinity causes osmotic stress, ion stress, and alterations of cellular ionic ratios, thus affect cellular metabolism [37]. For instance, in *Amphora subtropica* and *Dunaliella* sp., chlorophyll, primary carotenoid, and protein contents were highest under optimal salinity for cell growth [81]. Similarly, phycobiliprotein content was higher under the salinity condition that is desirable for cell growth [80]. However, the accumulation of storage lipids and carbohydrates was enhanced under low salinity stress in *Dunaliella tertiolecta* [82]. In some microalgae, the accumulation of storage lipids (long-term energy product) increased under salinity stress condition by channeling carbon from starch (short-term energy reserve) through the upregulation of lipid biosynthesis and downregulation of starch biosynthesis [36,37]. On the other hand, the accumulation of secondary carotenoids, such as β-carotene, enhanced under salinity stress [83], which can be attributed to the generation of oxidative stress [84]. Hence, suitable salinity is crucial for the biosynthesis of primary metabolites, including chlorophylls, primary carotenoids, and proteins, while the accumulation of storage lipids and secondary carotenoids increases under salinity stress condition.

### 3.2. Co-Production of Multiple Compounds under Nutrient Conditions

#### 3.2.1. Carbon

Carbon is the basic element of most metabolites in marine microalgae. In autotrophic cultivation, CO_2_ and bicarbonate (HCO_3_^−^) are the carbon sources absorbed by marine microalgae, and HCO_3_^−^ is converted into CO_2_ at the action of carbonic anhydrase [85,86]. Generally, adequate carbon concentration is essential for cell growth and metabolite accumulation. For example, biomass and the contents of chlorophyll and carotenoid increased with the increase in CO_2_ concentration in *Microchloropsis gaditana* NIES 2587 [87] and *Thalassiosira pseudonana* [88]. However, under excessively high CO_2_ concentration, storage lipid content increases by decreasing protein and carbohydrate contents [89]. This trend can be attributed to the promotion of carbon fixation by high CO_2_ concentration, leading to more precursors, energy, and reductants for the biosynthesis of these metabolites [90]. Moreover, the accumulation of secondary carotenoids, as well as storage carbohydrates and lipids, increase under excessive CO_2_ and/or HCO_3_^−^ concentration for handling the low pH condition. Therefore, in phototrophic marine microalgae, the contents of chlorophylls, primary carotenoids, proteins, structural carbohydrates, and membrane lipids increase under optimal carbon source, whereas the accumulation of secondary carotenoids, as well as storage carbohydrates and lipids, are enhanced under excessive carbon source.

#### 3.2.2. Nitrogen

Nitrogen is mostly consumed in the form of ammonium (NH_4_^+^) or nitrate (NO_3_^−^) by microalgae. Sufficient nitrogen is essential for cell growth and the accumulation of primary metabolites, while it is not suitable for the biosynthesis of secondary metabolites and storage lipids and carbohydrates. It was found that fucoxanthin and DHA contents enhanced with the increase in nitrate concentration from 25 to 100 mg/L in *Isochrysis* strain CCMP1324 [27]. Similarly, phycoerythrin content was boosted when nitrate concentration was increased from 800 to 1100 and 1320 μΜ in *Rhodomonas* sp. [76]. On the other hand, excessive nitrogen can either reduce the accumulation of primary metabolites or have no effect at all. For instance, lutein content decreased when nitrogen concentration exceeded 1000 mg/L in *Chlamydomonas* sp. JSC4 [91], while it stabilized at 9.65 mg/g under excessive nitrogen concentration in *Chlorella sorokiniana* FZU60 [92]. At the other end, nitrogen starvation induces the accumulation of storage carbohydrates and lipids, which may be due to the transformation of protein or peptides into these energy-rich metabolites [93]. It was reported that the accumulation of lipids increased with decreased proteins and carbohydrates when nitrogen was depleted in *P. tricornutum* [94]. Furthermore, nitrogen depletion is an efficient strategy to induce secondary metabolite accumulation. For instance, β-carotene content was significantly induced under nitrogen starvation in *D*. *salina* [21]. Based on these observations, the accumulation of primary metabolites, such as chlorophylls, proteins, lutein, and fucoxanthin, increase under nitrogen sufficient condition, while storage carbohydrates and lipids, as well as secondary metabolites contents, enhance under nitrogen-depleted condition.

#### 3.2.3. Phosphorus

Phosphorus is an important component of nucleic acids (DNA and RNA) and phospholipids (element of cell membranes) in microalgae, and plays an essential role in energy transfer in the form of ATP [63]. This element is assimilated in the form of phosphates (PO_4_^3−^) by microalgae. It is commonly recognized that sufficient phosphorus is required for cell growth and production of primary metabolites in microalgae because increasing phosphorus assimilation is beneficial for the biosynthesis of nucleic acids and phospholipids, and may further affect the accumulation of most primary metabolites [95]. For instance, the contents of DHA and fucoxanthin enhanced with the increase in phosphorus concentration from 1.13 to 4.5 mg/L in the *Isochrysis* strain CCMP1324 [27]. Carbohydrate content was found to be increased with the rise in phosphorus concentrations from 0 to 120 mg/L in *Rhodosorus* sp. SCSIO-45730 [31]. The accumulation of phycobiliproteins requires sufficient phosphorus because it is degraded under phosphorus starvation conditions [96]. Protein accumulation was not significantly affected under different phosphorus concentrations in *Chaetoceros muelleri* [97], which may be because phosphorus is not a main component of proteins. On the other hand, similar to nitrogen starvation, phosphorus limitation has been widely used to produce various high-value secondary metabolites and storage lipids and carbohydrates. It has been reported that lipid content, especially TAG levels, increased under phosphorus depletion condition in *Nannochloropsis* sp. PJ12 [98], the starch content increased under a phosphorus-deprived condition in *Tetraselmis subcordiformis* [99], and the biosynthesis of β-carotene and TAG enhanced under phosphate deprivation condition in *D. salina* [100]. Hence, it has been established that sufficient phosphorus availability is desirable for most primary metabolites accumulation, while phosphorus limitation is essential to induce the biosynthesis of secondary metabolites and storage lipids and carbohydrates.

#### 3.2.4. Sulfur

Sulfur (S) is a crucial element of protein as it exists in some essential amino acids, such as cysteine and methionine. Particularly, the disulfide bridges, constituting cysteine-cysteine disulfide covalent bonds, play an essential role in the assembly and structure of proteins [63]. Sulfur is typically utilized by microalgae in the form of sulfate (SO_4_^2−^). Sufficient sulfur is important for primary metabolites accumulation, while sulfur limitation can induce the accumulation of secondary metabolites, as well as storage lipids and carbohydrates. For example, it was found that protein content enhanced when the sulfur ratio of the medium increased in *Nannochloropsis gaditana* [101]. However, starch content was significantly enhanced under sulfur-limited conditions in *Tetraselmis subcordiformis* [102], and the accumulation of β-carotene was improved during sulfur starvation in *D. salina* [103]. Thus, the sulfur limitation can be used to co-produce secondary metabolites, as well as storage lipids and carbohydrates, such as secondary carotenoids, neutral lipids, and starches.

### 3.3. Cultivation Strategies for Enhanced the Co-Production of Multiple Compounds

Environmental and nutrient conditions significantly affect the accumulation of microalgae-derived metabolites, and each factor shows distinct effects. For the co-production of multiple metabolites, various conditions should be considered and comprehensively applied. Hence, numerous cultivation strategies based on environmental and nutrient conditions (Table 2) can be explored to enhance the co-production of multiple compounds.

#### 3.3.1. Cultivation Strategies Based on Environmental Conditions

The biosynthesis of metabolites and cell growth are two important factors that need to be considered when microalgae are cultured for multiple compounds co-production, as they can affect the productivity of specific metabolites. However, some metabolites, especially secondary metabolites, showed an inverse trend with cell growth as the change of environmental conditions. Thus, two-stage cultivation strategies have been explored to enhance cell growth in the first stage and induce metabolite accumulation in the second stage [114]. A temperature decreasing strategy has been applied to lutein production in *Chlamydomonas* sp. JSC4 [75]. Similarly, a salinity-shift strategy has been applied to biodiesel production in *D. salina* KSA-HS022 [104] and lutein production in *Chlamydomonas* sp. JSC4 [91].

#### 3.3.2. Cultivation Strategies Based on Nutrient Conditions

Nutrients are crucial for microalgal growth and the accumulation of some metabolites, especially primary metabolites. A decrease in nutrient concentration during the cultivation process can limit the co-production of multiple compounds. Hence, semi-continuous and fed-batch strategies have been extensively applied to avoid the limitation or inhibition of substrates during microalgal cultivation. The productivities of DHA and fucoxanthin of *Isochrysis* strains CCMP1324 were significantly enhanced in a semi-continuous strategy [27]. Further, a semi-continuous cultivation strategy with urea limitation was applied to improve lipid production in *Chlorella* sp. [105]. In addition, fed-batch cultivation has been widely applied as an effective strategy in microalgal culture to improve cell growth and accumulation of high-value products, such as carbohydrates, proteins, lipids, and carotenoids. For instance, a fed-batch strategy with phosphate addition was applied to improve the co-production of lipid and chitin nanofibers in *Cyclotella* sp. [106]. Similarly, a nitrate fed-batch strategy significantly boosted biomass growth and phycocyanin production in *Arthrospira platensis* [107].

On the other hand, the accumulation of some metabolites, such as secondary metabolites, as well as storage carbohydrates and lipids, are enhanced under nutrient-starved conditions. Thus, a two-stage cultivation strategy, with sufficient nutrient for cell growth in the first stage, and nutrient limitation for metabolites accumulation during the second stage, has been used to promote the co-production of multiple compounds [114]. For example, a nitrogen sufficient/deficient strategy was used to increase TAG accumulation in *Isochrysis zhangjiangensis* [109] and *P. tricornutum* [110]. In addition, nutrient sufficient/deficient strategy has been widely used in secondary carotenoid production, such as β-carotene [108].

#### 3.3.3. Multiple Factors Integrated Cultivation Strategies

Cellular metabolism is affected by various environmental and nutrient factors during microalgal cultivation. Thus, multiple factors integrated strategies can be used to enhance the co-production of multiple compounds. The environmental factor-integrated strategy is an efficient way to promote metabolite accumulation. It was found that temperature and light decreasing strategy was effective for EPA and biodiesel production in *Nannochloropsis* sp. [111]. In addition, the nutrient factor-integrated strategy can be used to improve the accumulation of metabolites. A two-stage strategy with a combination of nitrogen and sulfur limitation significantly enhanced starch and carbohydrate production in *Chlorella salina* [112].

Moreover, environmental and nutrient factor-integrated strategy can be another good option to enhance the accumulation of metabolites. The combination of salinity and nitrogen depletion in a two-stage cultivation strategy was applied to improve lipid production in marine microalgae *Chlamydomonas* sp. JSC4 [113]. Additionally, a two-phase strategy, with short nitrogen starvation in phase-one followed by high light exposure in phase-two, was effective in obtaining optimal co-production of protein and carotenoid in *D. salina* [13].

Thus, various cultivation strategies, according to the features of metabolites in specific microalgae, can be used as an efficient and promising approach to improve the co-production of multiple compounds. However, to date, limited studies have been reported with respect to exploring various strategies for the co-production of multiple compounds. Furthermore, although strategies based on single environmental or nutrient factors have been widely carried out in microalgae cultivation, there is a scarcity of studies about environmental and/or nutrient factor-integrated strategies. Hence, additional efforts are required to explore and develop multiple strategies for the co-production of multiple compounds.

## 4. Biorefinery from Marine Microalgal Biomass

Over the past decades, extraction processes of multiple compounds, whether simultaneous or sequential, have been optimized for different raw materials. For instance, milk has been used to produce cheese and whey protein [115], sugarcane has been used to produce soluble sugars and the natural polymers [116], and soybean has been used to produce soybean oil and soy protein [117]. Compared to these raw products materials, marine microalgae are relatively novel feedstock for a biorefinery process. Hence, exploring efficient biorefinery systems in marine microalgae will help to reduce the cost and achieve the goal of circular bioeconomy.

**Figure 2:**
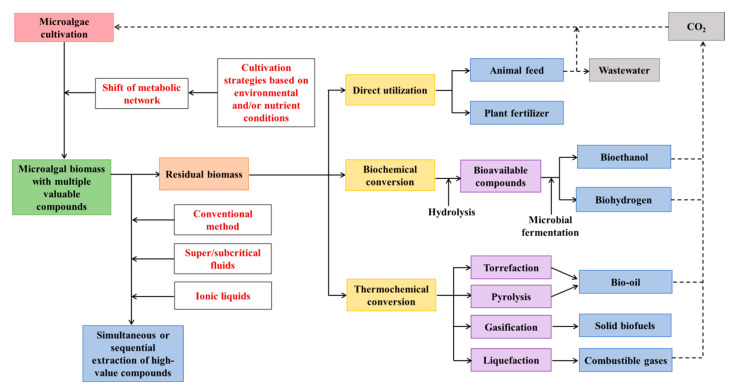
Proposed comprehensive utilization of marine microalgae for enhanced co-production of multiple compounds as a circular bioeconomy.

The development of biorefinery systems will depend on several parameters, including the amount of each metabolite in the specific microalgae, the market value of the metabolite, and cost and ease of extracting and/or purifying the metabolite from the crude extract. Hence, biorefinery systems can start with the co-extraction of high-value metabolites from marine microalgae, and remaining biomass can be further utilized or converted into valuable products (Figure 2). Table 3 lists the simultaneous or sequential extraction of multiple compounds from different marine microalgae species.

### 4.1. Co-Extraction of Multiple Compounds from Marine Microalgae

#### 4.1.1. Co-Extraction of Multiple Compounds from Marine Microalgae by Conventional Methods

Conventional methods of extraction can be categorized into physical and chemical (including enzymatic) methods. Vegetable oils have been commercially extracted from their respective feedstocks by means of an expeller or an oil press followed by solvent extraction. In addition, pretreatment methods via thermal or enzymatic treatments have been used to facilitate the penetration of solvent molecules [127]. However, these methods cannot be used for some marine microalgae, due to the rigid cell walls [128]. Moreover, small cell size and elastic cell walls of marine microalgae make cells easily squeeze through a mechanical device without complete cell disruption [129]. These are the main hindrances for industries to commercially produce pure or crude extracts from marine microalgae.

Numerous physical extraction techniques have been employed with marine microalgae biomass, including sonication, high-pressure homogenization, bead beating, microwave-assisted, and autoclave-assisted extractions. In addition, various chemical methods have been extensively studied, which includes the use of several common organic solvents, such as hexane, ethanol, chloroform, and methanol. Acid and alkaline solutions have also been used to either precipitate or solubilize large biomolecular metabolites such as proteins. Similarly, enzymes (trypsin, cellulase, and glucosidase) have been used for the extraction of microalgal compounds. In general, physical extraction is normally used as a pretreatment of chemical extraction, since it is difficult to obtain a specific compound.

Chemical extraction techniques have been widely used in metabolite extraction from marine microalgae, which are easy to achieve simultaneous or sequential extraction of multiple compounds. The chemical properties of the compounds of interest are important to simultaneously or selectively extract them from microalgal biomass. Both lipids and pigments (carotenoids and chlorophylls) are lipophilic, and thus, organic solvent extraction is a conventional and widely used method to isolate bioactive compounds from marine microalgae. On the other hand, proteins and carbohydrates are water-soluble, which are usually extracted by aqueous acidic or alkaline solutions. Thus, most biorefinery processes focus on the separation of nonpolar lipids and pigments from polar proteins and carbohydrates. Since marine microalgae were originally studied for their potential as biofuel feedstocks, most of the proposed biorefinery systems start with the extraction of oil. However, due to the highly similar polarity of lipids and pigments, these compounds are simultaneously extracted in organic solvents. Thus, most crude microalgal oil appears green in color, due to the presence of chlorophylls. Similarly, extracted oils from microalgal species, such as *D. salina*, with a high content of carotenoids appear red and orange, due to the high amounts of beta-carotene. Separation of high-value carotenoids requires numerous chromatographic techniques or optimization of solvent polarity using solvent mixtures. It was reported that fucoxanthin and EPA could be purified and concentrated from *P. tricornutum* through a series of extraction and separation processes [11]. In addition, a two-phase system was proposed to separate the biodiesel from free lutein [15].

In some cases, lipid extraction may not be the best initial step in the biorefinery process. It was shown that lipid extracted microalgal biomass had low protein yields compared to that of whole biomass [130]. The difference in the yields may be due to the denaturing of proteins during solvent extraction, thereby reducing their solubility. Perhaps, it may not be the best method to solubilize the proteins for isolation. A three-phase partitioning method was developed to separate lipid, polysaccharide, protein, and chlorophyll from wet *Chlorella* spp. [131]. The 40% (NH_4_)_2_SO_4_ (wt, %) solution and t-butanol were used to separate protein and polysaccharide in the supernatant, and residual biomass was resuspended in ethanol/n-hexane (1:1, *v*/*v*) with sulfuric acid to separate the lipids and chlorophylls.

In general, conventional extraction methods involve multi-step processes with each step utilizing various organic and/or aqueous solvents. The entire procedure can be quite laborious, increasing production costs. Moreover, although organic solvents have been extensively applied to the extraction of microalgal lipids for biodiesel production, they may lead to contamination of the final products; thus, limiting the consumer market [132]. Green solvents, such as ionic liquids and supercritical liquid extraction, have been proposed as alternative methods to recover bioactive components from microalgae [133,134].

#### 4.1.2. Co-Extraction of Multiple Compounds from Marine Microalgae by Using Super/Subcritical Fluids

When a substance is placed in a condition above its thermodynamic critical temperature and pressure, it becomes a supercritical fluid. On the other hand, subcritical fluids are substances that are pressurized heated to between their boiling points and the critical points so that the substances remain in the liquid state. Extractions using supercritical and subcritical fluids follow similar principles as traditional organic solvent extraction methods except that the extraction time is greatly reduced because of the high diffusivity and low viscosity of the fluids.

Supercritical liquid extraction has attracted wide attention because of its high-speed extraction, ease of solvent separation from the extracts, and low operating temperature [135]. CO_2_ is commonly-used for supercritical liquid extraction, due to its low toxicity and low critical temperature (31 °C) [136]. Thus, supercritical CO_2_ (ScCO_2_) has been extensively studied to extract bioactive components such as beta-carotene [137], lutein [138], and fatty acids [139] from marine microalgae. Most studies focused on extracting single products mainly because the separation of compounds with similar polarities (e.g., fatty acids and pigments) is rather costly and complicated.

The extraction efficiency of ScCO_2_ can be affected by various operating parameters, such as temperature, pressure, duration, and extraction cycles. In addition, suitable pretreatment of feedstock and the use of co-solvents can increase the extraction efficiency [140]. Moreover, the density of ScCO_2_ can influence its solubility for different compounds. It was demonstrated that the change in ScCO_2_ density affected the type of lipids extracted according to the fatty acid chain length and degree of saturation [141]. Therefore, the polarity of ScCO_2_ can be tuned to extract specific products by varying the operating parameters [142].

The extraction of compounds by ScCO_2_ can also be modified by using a co-solvent. Ethanol is an effective co-solvent for the supercritical extraction of hydroxy-carotenoids from different matrices, due to its ability to increase the solubility of hydroxy-carotenoids in ScCO_2_ [143]. In addition, ethanol is a green solvent, and trace ethanol residues in final extracts do not compromise the application of products in nutraceutical or pharmaceutical industries [144]. It was used as a co-solvent to extract carotenoids and chlorophylls from three different marine microalgae (*Nannochloropsis gaditana*, *Synechococcus* sp., and *D. salina*), and high yields of the two compounds were obtained [143].

Previous studies show that ScCO_2_ was selective for neutral lipids, such as TAG, but was not sensitive to polar lipids, which indicates that ScCO_2_ is highly nonpolar [145]. Thus, to improve the polarity of the pressurized liquid, other studies investigated the use of subcritical liquids. It was shown that subcritical dimethyl ether could extract carotenoids and lipids from wet microalga biomass easily because of the partial miscibility of liquified dimethyl ether with water [146]. Another interesting subcritical liquid is subcritical water. In normal atmospheric conditions, water is considered as a good solvent for extracting polar compounds. However, in subcritical conditions, the dielectric constant of water decreased from 80 to 33, which is similar to organic solvents like ethanol and methanol [147]. It was shown that bio-oil could be extracted from *N. salina* using subcritical water [148]. Phenolic compounds, such as caffeic acid, ferulic acid, and *p*-coumaric acid, were also successfully extracted from *Chlorella* sp. using subcritical water [149].

The use of supercritical/subcritical fluids is quite promising for the co-production of multiple compounds, mainly due to the ease of separating the solvent from the compounds of interest, which is one of the labor- and energy-intensive steps in conventional methods. The use of pressure greatly reduces the extraction time, making the method more cost-effective than the traditional organic solvent extraction methods. Moreover, the operating parameters are easier to manipulate compared to testing different combinations of solvent mixtures, as in the case of the conventional methods. However, the main disadvantage of using supercritical/subcritical fluids is the high capital costs, especially in large-scale extractions. Nevertheless, in the near future, the cost of these high-pressure extractors will surely be declined as this technique has the potential to be widely used in the pharmaceutical industries.

#### 4.1.3. Co-Extraction of Multiple Compounds from Marine Microalgae by Using Ionic Liquids (ILs)

ILs are salts, typically consisting of bulky organic cation or anion, with melting points below 100 °C [150]. They exhibit several properties, such as low flammability, low vapor pressure, and thermal stability, thus, making them attractive alternatives to the existing laboratory solvents [151]. Common cations are imidazolium, pyridinium, ammonium, and phosphonium derivatives, while the anions are simple halides and bulky organic ions, such as trifluoromethanesulfonate and tosylate. Numerous studies have shown that ILs can replace chloroform in the conventional Bligh and Dyer’s method for extracting lipids from microalgae [152,153,154]. The solubility of biopolymers in ILs has been considered as the reason for their extraction efficiencies [119,155,156]. This IL property means that the cellular components can be simultaneously released for further downstream processing and purification. In fact, a number of studies have shown that ILs are effective solvents for pretreatment of wet microalgal biomass. For example, full deconstruction of the wet biomass was observed after heating at 100–140 °C for <50 min in the presence of ILs [157]. Thus, the use of ILs improves the energy efficiency of the biorefinery process, as there is no need to spend more energy in drying the biomass before the extraction. Aside from using ILs as a pretreatment method, ILs can be used as solvents for the separation of different microalgae products. The IL Iolilyte 221PG, when mixed with citrate buffer, could separate pigments from proteins of *Neochloris oleoabundans* [158]. Moreover, it has been reported that the changes in cation and anion greatly affect the extraction efficiency [152,155]. Thus, similar to supercritical/subcritical fluids, ILs can be tuned to extract specific compounds by varying the cations and anions [159]. Further research is required to assess the different combinations of cations and anions for the simultaneous or sequential extraction of high-value products.

From the aforementioned studies, it is clear that the use of ILs in a microalgal biorefinery process has the advantage of having a combined cell disruption, extraction, and purification step to produce either a single compound or a product with multiple compounds. Nevertheless, amidst all the advantages of ILs, the toxicity of these substances needs to be further assessed. Previous studies have reported the non-toxic properties of ILs, but most of these studies were based on microorganisms, which needs to be validated, especially if the IL-extracted product is used for human consumption [160]. It is proposed that the potential of ILs to be used in the food industry can be achieved by using a combination of naturally sourced cations and anions, such as cholinium and amino acids [159]. Future policies with regards to the use of ILs in the food industry will greatly affect the fate of the method in algal biorefineries.

### 4.2. Utilization and Conversion of Microalgal Residues after Extraction (MRAE)

After the extraction of high-value compounds (basically lipids and pigments, in some cases proteins) from biomass, remaining microalgal residues are still valuable for other applications. The residual biomass can be directly utilized or can be converted into another form of high-value compounds (Figure 2).

#### 4.2.1. Direct Utilization of MRAE

If proteins are not extracted from marine microalgal biomass, then MRAE can be used as animal feed. MRAE can replace a certain proportion of corn and soybean meal in the diets of poultry, pig, and fish, and there was no significant negative effect on growth performance and health of animals [161]. It was found that a defatted microalgae meal (*Nannochloropsis* sp.) can be used (15% of the diet) to replace fish meal in the test diet without compromising the performance of the European sea bass [162].

In addition, MRAE rich in protein and other nutrients can be utilized as biofertilizer for sustainable plant production. MRAE of two microalgal species (*Chlorella variabilis* and *Lyngbya majuscula*) was utilized to substitute for the chemical nitrogen fertilizer requirement of maize (*Zeamays* L.) crop in different proportions [163]. The grain yield obtained using MRAE was equivalent to those cultivated with chemical fertilizers, and no detrimental effects were observed in soil properties were found. Hence, MRAE could be employed to reduce the usage of chemical fertilizers, thus promoting maize crop production in a sustainable manner. However, after the extraction of marine microalgae, residues of some toxic compounds, such as residual salinity or organic solvents may have negative effects on plant growth and production, which should be carefully considered prior to utilization.

#### 4.2.2. Biochemical Conversion of MRAE

The proteins and carbohydrates in MRAE can be converted into bioavailable compounds by hydrolysis, and these hydrolysates can be further used as potential substrates for microbial fermentation to produce valuable products, such as bioethanol, biohydrogen, and methane [164]. The pretreatment of MRAE by thermal, chemical, enzymatic, and mechanical hydrolysis can significantly improve its digestibility for the microorganism. Thermal hydrolysis can significantly enhance the solubility of microalgal cells due to the partial dissolution of hemicellulose and cellulose at higher temperatures (>120 °C) [165]. Acidic hydrolysis and alkaline hydrolysis are two conventional chemical pretreatments for marine microalgae. Acid treatment is effective to dissolve carbohydrates, while alkali treatment is beneficial to dissolve proteins [166]. In addition, enzymes can be used to make the MRAE easily digestible [18]; however, this method may potentially increase the operating cost. Moreover, MRAE can be mechanically hydrolyzed using hydrodynamic cavitation and other methods [167]. After the pretreatment of the MRAE, the resulting product can be used for various applications. For example, saccharified MRAE was used as a substrate for *Saccharomyces cerevisiae* to produce bioethanol [168]. Biogas, such as methane, also has been produced by anaerobic fermentation of different MRAE [169].

#### 4.2.3. Thermochemical Conversion of MRAE

MRAE can also be decomposed into solid, liquid, and gas biofuels through thermochemical conversion methods, including torrefaction, liquefaction, pyrolysis, and gasification. The main product of liquefaction and pyrolysis is bio-oils, whereas solid biofuels and combustible gases are the main product of torrefaction and gasification, respectively [170]. Compared to biochemical conversion, thermochemical conversion can convert MRAE into biofuels in a short time (several minutes to several hours). However, relatively high pressure and temperature are required for this process.

In torrefaction, biomass is thermally degraded in an inert environment at a temperature range of 200–300 °C. It is also known as mild pyrolysis because of its low operating temperatures [171]. The properties of fuels prepared from microalgal residues by torrefaction were investigated, and it was found that the Hardgrove Grindability Index (HGI) of torrefied microalgal residue was 48.53 when the torrefaction temperature was 250 °C, which was higher than that of sub-bituminous coal [172]. Residues of *Chlamydomonas* sp. JSC4 and *Chlorella sorokiniana* CY1 were subjected to torrefaction with nitrogen gas at 200–300 °C, and the results showed that biomass torrefaction at 300 °C for a short duration was an energy-saving route to upgrade biomass when compared to that for 60 min at lower temperatures [173].

In pyrolysis, biomass is putrefied in the absence of oxygen at high temperatures (400–600 °C). MRAE from different algal species (such as *Chlamydomonas*, *Chlorella sorokiniana*, and *Dunaliella tertiolecta*) have been subjected to pyrolysis [125,174]. The catalyst involved in pyrolysis (catalytic pyrolysis) can improve the quality of bio-oils produced from MRAE and reduce the reaction temperature to as low as 300 °C. In addition, microwave-assisted heating has been introduced in the pyrolysis of MRAE [175].

Gasification is one of the most efficient technologies used for the conversion of lignocellulosic biomass. In gasification, biomass is converted into H_2_, CO, CH_4_, and other combustible gases in an insufficient oxidizer environment at high temperatures (>700 °C) without combustion [170]. Gasification using MRAE of marine microalgae has been reported [176]. Supported-metal catalysts are effective for improving the gasification process of lignocellulosic biomass. For example, the maximum hydrogen yield reached 413 cc/g algae for *Nannochloropsis oculata* when using 10% Fe_2_O_3_–90% CeO_2_ as a catalyst [176].

Liquefaction is a thermal process that can convert biomass into liquid fuel at high temperatures (250–350 °C) and pressures (5–20 MPa), and it is suitable for MRAE with high moisture [170]. MRAE of different species (*Spirulina*, *Nannochloropsis,* and *Chlorella*) have been applied to produce biofuel through the liquefaction process [177,178]. Either homogenous or heterogeneous catalysts can be used to facilitate liquefaction reactions. Sodium carbonate is the most commonly used homogenous catalyst for microalgae liquefaction. Biochemical components from microalgae were liquefied with or without catalyst (1 M Na_2_CO_3_), and it was found that both proteins and lipids can be efficiently converted into bio-oil without catalysts, while carbohydrates were best processed by using Na_2_CO_3_ [177]. In addition, it has been reported that water at subcritical conditions is an effective solvent for liquefaction of MRAE, which undergoes hydrolysis (or depolymerization) and repolymerization in biomass, and also transforms the biomass into bio-oil, gas, and solid compounds.

## 5. Conclusions and Future Outlook

Marine microalgal cells can simultaneously accumulate various valuable compounds; thus, the co-production of multiple products is achievable. The biosynthesis of lipids, pigments, carbohydrates, and proteins, is highly interconnected in the metabolic network and controlled by limiting steps. Metabolic flux may shift to different metabolites under specific cultivation conditions. Exploring novel cultivation strategies by integrating environmental and/or nutrient factors can be an effective way to improve the co-production of multiple compounds. Additionally, efficient biorefinery techniques for simultaneous or sequential extraction of multiple products, followed by utilization or conversion of microalgal residues to high-value products, will enhance the economic feasibility of marine microalgae production in large industries.

However, to achieve the goal of comprehensive utilization of marine microalgae for enhanced co-production of multiple compounds, several challenges need to be addressed. Above all, the ability of co-production of multiple compounds in marine microalgae is species-specific. Thus, the cell composition and optimal cultivation condition should be investigated for each microalgal species. Moreover, new cultivation strategies need to be explored to enhance the co-production. In addition, current biorefinery techniques in large industries are still energy and labor-intensive, which are not conducive for environmental and economic sustainability. Hence, green and low-cost biorefinery techniques need to be developed to extract all the bioactive components of microalgal cells. Besides, compounds with the same polarity may be difficult to be separated after co-extraction. Therefore, it is imperative to develop efficient separation techniques after the co-extraction of multiple compounds. Hence, the possibility of sequential extraction is of great interest to improve the sustainability of the biorefinery system. Moreover, the use of biofuel produced from MRAE will generate greenhouse gas CO_2_, which can be used as an inorganic carbon source for marine microalgae growth. Similarly, the wastewater from farmed animals fed by MRAE can be applied to marine microalgae cultivation. Hence, circular bioeconomy can be achieved by the co-production of multiple compounds (Figure 2).

## Figures and Tables

**Table 1:** List of marine microalgae species for the co-production of multiple compounds.

Category	Microalgae Species	Metabolites	References
Chlorophyta	*Chlamydomonas* sp. JSC4	Fatty acid (20.256%)	[19]
		Lutein (0.382%)	
		Carbohydrate (~15%)	
		Protein (~40%)	
	*Chlamydomonas* sp. KNM0029C	Biodiesel (15.6%)	[20]
		Bioethanol (14.4%)	
		Protein (24.2%)	
	*Dunaliella salina*	Protein (419 pg/cell)	[13]
		Carotenoid (22 pg/cell)	
	*Dunaliella salina*	Triglyceride (~8%)	[21]
		β-carotene (~11%)	
	*Chlorella* sp. AE10	Carbohydrate (75.9%)	[22]
		Lipid (15.53%)	
		Lutein (0.958%)	
	*Stichococcus* sp.	Carbohydrate (40.63%)	[23]
		Protein (26.25%)	
		Lipid (11.56%)	
	*Tetraselmis* sp. CTP4	Lutein (0.317%)	[24,25]
		β-carotene (0.321%)	
		Protein (~40%)	
		Lipid (~5%)	
		Carbohydrate (~45%)	
Chrysophyta	*Isochrysis zhangjiangensis*	Fucoxanthin (2.329%)	[26]
		Stearidonic acid (~2%)	
		Pigment (~5%)	
		Lipid (~25%)	
		Protein (~25%)	
		Carbohydrate (~25%)	
	*Isochrysis* sp. CCMP1324	Fucoxanthin (1.41%)	[27]
		DHA (1.71%)	
Bacillariophyta	*Phaeodactylum tricornutum*	Fucoxanthin (~0.6%)	[12]
		EPA (~4.8%)	
		Lipid (~40%)	
	*Cylindrotheca fusiformis*	Fucoxanthin (~0.5%)	[12]
		EPA (~2.1%)	
		Lipid (~40%)	
	*Nitzschia laevis*	Fucoxanthin (1.56%)	[28]
		EPA (3.43%)	
		Lipid (~35%)	
	*Thalassiosira weissflogii*	Fucoxanthin (0.95%)	[29]
		EPA (7.45%)	
		Lipid (45%)	
Heterokontophyta	*Nannochloropsis gaditana*	EPA (~4%)	[30]
		Carotenoid (~1.3%)	
		Lipid (~24%)	
Rhodophyta	*Rhodosorus* sp. SCSIO-45730	Carbohydrate (43.8%)	[31]
		β-Glucan (19.4%)	
		Protein (~25%)	
		Lipid (7%)	
Cyanophyta	*Spirulina* sp.	Carbonic anhydrase (25.5 U/g)	[14]
		C-phycocyanin (9%)	
		Allophycocyanin (7%)	

**Table 2:** Cultivation strategies for multiple compounds production.

Category	Strategies	Microalgae Species	Metabolites	References
Strategies based on Environmental conditions	Temperature decreasing strategy	*Chlamydomonas* sp. JSC4	Lutein (3.25 mg/L/d)	[75]
	Salinity increasing strategy	*Dunaliella salina* KSA-HS022	Biodiesel (56.5 mg/L/d)	[104]
		*Chlamydomonas* sp. JSC4	Lutein (1.92 mg/L/d)	[91]
Strategies based on nutrient conditions	Semi-continuous strategy	*Isochrysis* strains CCMP1324	DHA (9.05 mg/L/d) Fucoxanthin (7.96 mg/L/d)	[27]
		*Chlorella* sp.	Lipid (139 mg/L/d)	[105]
	Fed-batch strategy	*Cyclotella* sp.	Lipid (31 mg/L/d) Chitin nanofibers (17 mg/L/d)	[106]
		*Arthrospira platensis*	Phycocyanin (~20 mg/L/d) Allophycocyanin (~10 mg/L/d)	[107]
	Nutrient sufficient/deficient strategy	*Dunaliella salina*	β-carotene (18.5 mg/L/d)	[108]
		*Isochrysis zhangjiangensis*	TAG (~90 mg/L/d)	[109]
		*Phaeodactylum tricornutum*	TAG (N/A)	[110]
Multiple factors integrated strategies	Temperature and light decreasing strategy	*Nannochloropsis* sp.	EPA (~10 mg/L/d) Lipid (~30 mg/L/d)	[111]
	Two-stage cultivation with a combination of nitrogen and sulfur limitation	*Chlorella salina*	Starch (~2 mg/L) Carbohydrate (~10 mg/L)	[112]
	Two-stage strategy with salinity and nitrogen depletion	*Chlamydomonas* sp. JSC4	Lipid (223.2 mg/L/d)	[113]
	Two-stage strategy with short nitrogen starvation/high light exposure	*Dunaliella salina*	Protein (22 mg/L/d) Carotenoid (3 mg/L/d)	[13]

**Table 3:** Simultaneous or sequential extraction of multiple compounds from marine microalgae.

Microalgae Species	First Extracted Products	Sequentially Extracted Products	References
*Chlorella* spp.	Carotenoids Chlorophylls	Proteins	[118]
	Lipids	Polysaccharides for bioethanol	[119]
*Nannochloropsis* spp.	Biofuel	Biohydrogen	[120]
	Omega-3 rich oil	Proteins	[121]
	Lipids pigments	Biohydrogen	[122]
*Spirulina* spp.	Proteins	Biomethane and biocrude oil	[123]
	Phycocyanin	Chlorophylls	[124]
*Dunaliella tertiolecta*	Lipids Carbohydrates	Bio-oils	[125,126]
*Phaeodactylum tricornutum*	Fucoxanthin	EPA then chrysolaminarin	[11]
*Isochrysis galbana*	Carotenoids Nonpolar lipids	Carotenoids, chlorophylls, mid to highly-polar lipids then proteins and sugars	[16]
